# The Light Chain 1 Subunit of the Microtubule-Associated Protein 1B (MAP1B) Is Responsible for Tiam1 Binding and Rac1 Activation in Neuronal Cells

**DOI:** 10.1371/journal.pone.0053123

**Published:** 2012-12-27

**Authors:** Daniel R. Henríquez, Felipe J. Bodaleo, Carolina Montenegro-Venegas, Christian González-Billault

**Affiliations:** Laboratory of Cell and Neuronal Dynamics (CENEDYN), Department of Biology, Faculty of Sciences, Universidad de Chile, Santiago, Chile; University of Birmingham, United Kingdom

## Abstract

Microtubule-associated protein 1B (MAP1B) is a neuronal protein involved in the stabilization of microtubules both in the axon and somatodendritic compartments. Acute, genetic inactivation of MAP1B leads to delayed axonal outgrowth, most likely due to changes in the post-translational modification of tubulin subunits, which enhances microtubule polymerization. Furthermore, MAP1B deficiency is accompanied by abnormal actin microfilament polymerization and dramatic changes in the activity of small GTPases controlling the actin cytoskeleton. In this work, we showed that MAP1B interacts with a guanine exchange factor, termed Tiam1, which specifically activates Rac1. These proteins co-segregated in neurons, and interact in both heterologous expression systems and primary neurons. We dissected the molecular domains involved in the MAP1B-Tiam1 interaction, and demonstrated that pleckstrin homology (PH) domains in Tiam1 are responsible for MAP1B binding. Interestingly, only the light chain 1 (LC1) of MAP1B was able to interact with Tiam1. Moreover, it was able to increase the activity of the small GTPase, Rac1. These results suggest that the interaction between Tiam1 and MAP1B, is produced by the binding of LC1 with PH domains in Tiam1. The formation of such a complex impacts on the activation levels of Rac1 confirming a novel function of MAP1B related with the control of small GTPases. These results also support the idea of cross-talk between cytoskeleton compartments inside neuronal cells.

## Introduction

In cultured hippocampal neurons, axon outgrowth and the concomitant breaking of symmetry proceed through a series of stereotyped events [1]. Shortly after plating, spherical neuronal cells develop several minor processes of the same length. After a few hours in culture, one of these processes, with a large and highly dynamic growth cone, starts to grow, generating a polarized cell [2], [3]. The remaining minor processes, which grow more slowly, will subsequently become dendrites. Cumulative evidence shows that microtubules and actin microfilaments may reciprocally influence their dynamic behaviors, and are an important element supporting axon specification, guidance and elongation [3], [4]. In a seminal paper, Bradke and Dotti showed that the administration of cytochalasin D (an actin depolymerizing drug) promotes the formation of supernumerary axons [5], indicating that the tight regulation of actin filaments is necessary for determining a single axon. Amongst many other molecules, Rho GTPases are the master regulators of the actin cytoskeleton and have been implicated in diverse cellular processes such as cytokinesis, cellular adhesion and migration [6], [7]. More importantly, Rho GTPases have been reported to be key regulators of neurite extension and retraction, axon specification and polarization [3], [8].

Small GTPases act as molecular switches, cycling between an inactive, GDP-bound state and an active GTP-bound state. This cycling is regulated by proteins belonging to the guanine nucleotide exchange factor (GEF) and GTPase-activating protein (GAP) families [9], [10]. Once activated, Rho GTPases interact with effectors to propagate downstream signaling events that target multiple signal transduction pathways controlling various aspects of cell biology. The most-studied members of the Rho GTPase family are RhoA that regulates neurite collapse, and Rac1 and Cdc42 that induce extensive and protrusive actin polymerization leading to the formation of lamellipodia and filopodia, respectively [11]–[15]. Several Rho GTPase regulatory proteins are involved in neuronal polarization. One of them is the Rac1-specific GEF Tiam 1 (T-lymphoma and metastasis 1 protein) that has been identified as a Rac1 upstream regulator of neuronal polarity [16]–[19]. In hippocampal neurons, Tiam 1 accumulates in the axonal shafts and the growth cone of the prospective axon. Overexpression of Tiam1 induces multiple axon-like neurites, whereas the depletion of Tiam1 inhibits axon formation by preventing actin filament reorganization. Moreover, Tiam1 also associates with dynamic tyrosinated microtubules [18].

Previously, we showed that MAP1B (microtubule associated protein 1B) can regulate Rac1 activity during axonal outgrowth owing to its interaction with Tiam1 [20], [21], but the molecular domains involved in such interactions were not uncovered. MAP1B is the first MAP to be expressed strongly in the nervous system during embryonic development [22], [23]. The role of MAP1B in axonogenesis has been widely studied [24]–[26]. Thus, suppression of MAP1B with antisense oligonucleotides inhibits laminin-enhanced axon growth [25], and there is a significant delay in axon outgrowth and a reduced rate of axon elongation in cultured hippocampal pyramidal neurons from MAP1B-deficient mice [26]–[28].

MAP1B is a 320 kDa protein that is translated as a polyprotein which is subsequently cleaved into two subunits termed the heavy chain (HC), comprising the first 2,200 amino acids and the light chain 1 (LC1), which corresponds to the C-terminal 250 amino acids [29]. Both protein subunits form a macromolecular complex where HC and LC1 are the regulatory and active subunits, respectively [30].

MAP1B deficiency results in decreased Rac1 and Cdc42 activities and increased RhoA activity [20]. Interestingly, MAP1B deficiency also reduces the amount of tyrosinated microtubules, most likely due to an interaction between MAP1B and the tubulin tyrosine ligase (TTL) enzyme [31]. Tyrosinated microtubules strongly bind Tiam1, specifically at the growth cones of growing axons [18]. The mechanism involved in the MAP1B-Tiam1 interaction seems to be conserved in both young and adult neurons, where it may be involved in axon elongation and dendritic spine remodeling, respectively [20], [21]. These results suggest that the MAP1B-Tiam1 interaction may serve to regulate the cross-talk between actin and microtubules in developing neurons, affecting axon specification and elongation. In this work, we examine the molecular domains involved in the MAP1B-Tiam1 interaction, and show that PH-domains in Tiam1 are responsible for MAP1B binding. MAP1B binding to Tiam1 is promoted primarily by the LC1 subunit, leading to Rac1 activation, suggesting that the interaction between LC1 and Tiam1 is actually controlling the cross-talk between neuronal cytoskeleton compartments.

## Materials and Methods

### Primary antibodies

The following primary antibodies were used in this study: α-tubulin mAb (Sigma) diluted 1∶10000; FLAG mAb (Sigma) diluted 1∶4000; GST mAb (Sigma) diluted 1∶4000; a goat Ab against MAP1B diluted 1∶1000 (clone N-19, Santa Cruz Biotechnology); a rabbit Ab against Tiam1 diluted 1∶1000 (clone C-16, Santa Cruz Biotechnology); anti-HA mAb diluted 1∶1000 (F-7 clone, Santa Cruz Biotechnology); a rabbit Ab against c-myc diluted 1∶500 (clone A-14, Santa Cruz Biotechnology); a rabbit Ab against Rac1, diluted 1∶3000 (BD Bioscience).

### Plasmid constructs

Light chain 1 (LC1) of MAP1B (corresponding to amino acids 2100–2459) and the NTD (amino acids 2100–2336) and CTD (amino acids 2336–2459) fragments were amplified by PCR from a mouse embryonic brain cDNA preparation, digested with EcoRI/BamHI and subcloned into pGEX-6p1 (Promega). The same fragments were cloned into pcDNA3 for incorporation of a myc-tag). The following Tiam1 fragments were cloned: **T1**, N-terminal domain and PEST motif (amino acids 1–400); **T2**, N-terminal pleckstrin domain (N-PH, amino acids 400–740); **T3**, Ras binding domain (RBD) and PDZ domain (amino acids 740–1000); **T4**, Dbl-homology domain (DH, amino acids 1000–1263) and **T5**, C-terminal pleckstrin domain (C-PH, amino acids 1263–1591). PCR-amplified fragments were digested (T1, 2 and 3, BamHI/HindIII; T4, EcoRI/SalI; T5, BamHI/SalI) and subcloned into pCMV-Tag2 (Clontech) for addition of an N-terminal FLAG-epitope. All clones were confirmed by DNA sequencing. The cDNA encoding for MAP1B full length (**FL**; amino acids 1–2459), heavy chain (**HC**; amino acids 1–2185) and light chain 1 (**LC1**; amino acids 2210–2459) were cloned in the tetracycline (tTA) inducible expression vector pUHD10-3 for fusion to a C-terminal myc-tag (kindly provided by Dr. Friedrich Prospt (Austria) [30], [32]. The cDNA encoding for the N-terminal truncated Tiam1 **(C1199)** was cloned into a pcDNA3 vector containing a cytomegalovirus (CMV) promoter and a C-terminal HA-tag [33].

### Cell culture and transfection

COS7 and N1E115 cells were grown in DMEM medium containing 10% fetal bovine serum (FBS) and 1% penicillin/streptomycin in a humidified atmosphere of 5% CO_2_ at 37°C. For transfection, cells were seeded onto 100-mm or 60-mm dishes. The plasmids were transfected in serum-free OptiMEM medium using Lipofetamine 2000 (Invitrogene), according to the manufacturer's instructions. The medium was exchanged to the fresh serum-containing medium after 4 h. After transfection, cells were incubated for 48 h at 37°C.

### Rat hippocampal neuron primary culture

For immunofluorescence assays, cultures of rat hippocampal neurons were prepared as described previously by Kaech & Banker [34]. In brief, hippocampal cells from Sprague-Dawley rat embryos (E18.5) were dissociated after treatment for 20 min with 0.25% (w/v) trypsin (Gibco). Cells were plated at 1×10^4^ cells/cm^2^ in coverslips previously coated with poly-D-lysine 1 mg/ml (Sigma-Aldrich) in Neurobasal medium (Gibco) including 10% horse serum and Glutamine (Gibco). After 2 hours, the medium was replaced with Neurobasal medium supplemented with B27 and Glutamax (Gibco) in the absence of serum. Cells were kept in a humidified atmosphere of 5% CO_2_ at 37°C. Three days after plating, neurons were fixed for posterior analysis.

### Immunofluorescence

At 3 days *in vitro* (DIV), primary hippocampal neuronal cells were fixed with 4% paraformaldehyde, 4% sucrose for 30 min at 37°C and washed with phosphate-buffered saline (PBS). The cells were incubated with PBS, 0.1% Triton X-100 for 5 min and then blocked with PBS, 5% (w/v) bovine serum albumin (BSA) for 1 h. Subsequently, the cells were incubated with primary antibodies raised against the proteins indicated and diluted in PBS, 1% BSA. The fluorescent secondary antibodies (Molecular Probes, Invitrogen) were used at a dilution of 1∶400. Cells were analyzed on a Zeiss LSM510 Meta confocal scanning microscope.

### GST fusion protein preparation

For expression and purification of GST-LC1 and the Rac-GTP binding domain of GST-Pak1, BL21 (DE3) *E. coli* strains, carrying pGEX-LC1, pGEX-NTD-LC1, pGEX-CTD-LC1 and pGEX-PAK1 plasmids, respectively, were grown overnight (ON) in LB ampicillin medium. Overnight cultures were diluted 1∶100 and grown in fresh medium until OD_0.6_ at 37°C. Then, 0.1 mM isopropyl-β-D-thiogalactopyranoside (IPTG, final concentration) was added. Two h after induction, cells were collected and lyzed by sonication in lysis buffer A (50 mM Tris-HCl pH 8.0, 1% Triton X-100, 1 mM EDTA, 0.15 M NaCl, 25 mM NaF, 0.5 mM PMSF and 1× of protease inhibitor complex (Roche)). Cleared lysate was then purified by affinity with glutathione-Sepharose beads (Amersham). Loaded beads were washed ten times with lysis buffer B (lysis buffer A plus 300 mM NaCl) at 4°C. The GST fusion proteins were quantified and visualized in SDS-PAGE gels stained with Coomassie brilliant blue (CBBS) or immunoblotted using the anti-GST antibody.

### Protein-protein interaction pull down experiments

Glutathione sepharose loaded beads (40 µg) with GST-LC1 or LC1 subdomains (NTD-GST and CTD-GST) were incubated ON at 4°C with 1 mg of fetal brain lysates or COS7 cell lysates transfected with the FLAG-tagged Tiam1 constructs. Beads were washed six times with lysis buffer B, and the washed beads were suspended in SDS–PAGE sampling buffer. The bound proteins were subjected to immunoblot analysis. Beads loaded with GST alone were used as control.

### Immunoprecipitation

c-Myc antibody or FLAG antibody and the protein-A or G beads (Sigma) were incubated with N1E115, or COS7 cell lysates transfected with the indicated plasmids (coding FL, HC or LC1 of MAP1B and/or the FLAG-tagged construct of Tiam1) in lysis buffer A for 4 h at 4°C. Beads were washed three times with lysis buffer A and resuspended in SDS-PAGE sample buffer. Bound proteins were analyzed by immunoblot. High stringency conditions considered washing steps using buffer B (buffer A plus 300 mM NaCl) for 10 min each time. Beads were resuspended in SDS–PAGE sample buffer and bound proteins were analyzed by immunoblot.

### Rac1 activity pull down assay

The Rac1-GTP binding assay was performed essentially as described [35]. Briefly, the CRIB domain (amino acids 67–150) of p21-activated kinase (Pak1) that binds specifically to the Rac1-GTP but not to the inactive form of Rac1 (Rac1-GDP) was amplified and cloned into pGEX-CRIB-GST. Loaded beads were incubated for 1 h at 4°C with 1 mg of fresh COS7 cell lysates co-transfected with c-Myc-tagged MAP1B constructs and Tiam1 (C1199 HA-tagged) using fishing buffer (50 mM Tris-HCl pH 7.5, 10% glycerol, 1% Triton X-100, 200 mM NaCl, 10 mM MgCl_2_, 25 mM NaF, 1× protease inhibitor complex). The beads were then washed three times with washing buffer (50 mM Tris-HCl pH 7.5, 30 mM MgCl_2_, 40 mM NaCl) and the washed beads were suspended in SDS–PAGE sampling buffer. The bound Rac1-GTP was subjected to immunoblot analysis and quantified with respect to total Rac1 with the ImageJ program.

## Results

### Tiam1 interacts with light chain (LC1) but not with heavy chain (HC) of microtubule associated protein 1B (MAP1B)

MAP1B is essential for axonal elongation, most likely due to a dual role as a microtubule stabilizing factor [28], [36] and actin microfilament interacting protein [20], [37], [38]. We previously showed that neurons lacking MAP1B have abnormal localization of the guanine exchange factor (GEF) for Rac1, Tiam1 [20], which is accumulated in the cell body rather than concentrated at the most distal part of axons. We also showed that both proteins co-immunoprecipitate using either anti-MAP1B or anti-Tiam1 antibodies [20]. Since MAP1B possesses two different domains, termed the heavy chain (HC) and light chain 1 (LC1), in the present study we sought to characterize the molecular domains involved in the MAP1B-Tiam1 interaction.

**Figure 1:**
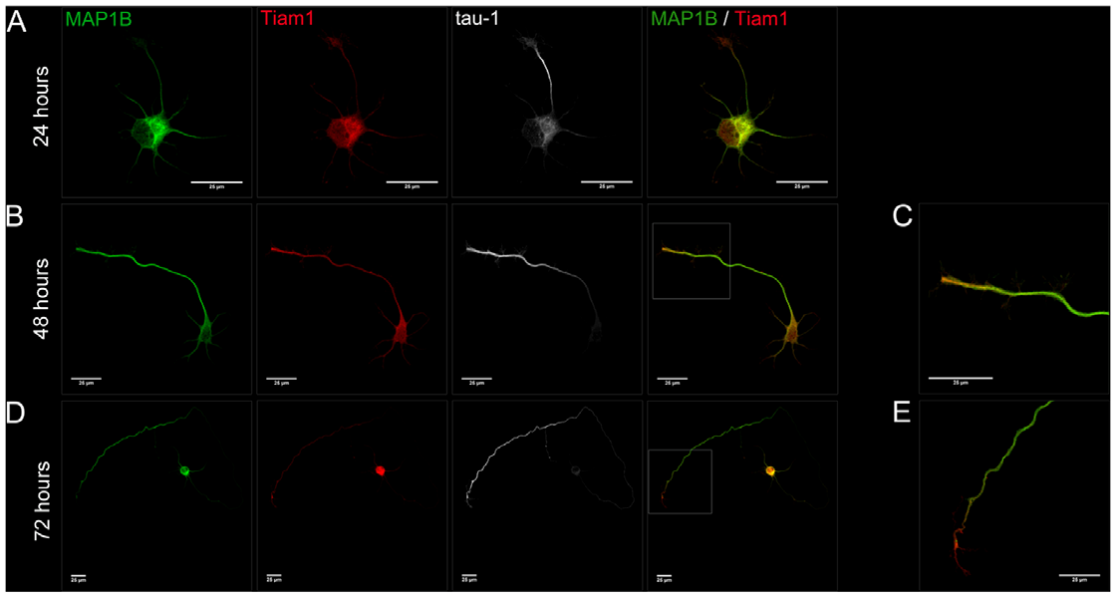
Tiam1 colocalizes with MAP1B and displays polarized distribution in hippocampal neurons during polarization. Hippocampal neurons grown for 24 (A), 48 (B) and 72 (D) hours in vitro and stained with MAP1B (green), Tiam1 (red) and axonal marker tau-1 (white). (C) and (E) shown a zoom of the MAP1B/Tiam1 merged channel (white square in B and D). Tiam1 and MAP1B are concentrated at the tip of the axon at 48 and 72 hrs in culture.

Therefore, we first performed an immunolocalization analysis of both MAP1B and Tiam1 in hippocampal neurons during polarization. As indicated in Figure 1, hippocampal neurons cultured for 24 hrs (Figure 1A), display one prospective axon, characterized by the presence of a bigger growth cone as compare with minor neurites. MAP1B and Tiam1 are present at the cell body and minor processes. However, the prospective axon exhibit MAP1B and Tiam1 staining, which begin to concentrate at the most distal part of the future axon. The identification of such minor neurite as the prospective axon is not only based on the growth cone size. The axonal marker tau-1 begins to accumulate in one of the minor neurites. At 48 hrs. we found that both proteins were still present at the somatodendritic and axonal compartments, but they were highly enriched at the most distal part of the axon (Figure 1B and 1C). Finally at 72 hrs, the staining at the somatodendritic compartment was faint, while the accumulation on the axonal tip persisted (Figure 1D and 1E). Interestingly, the expression pattern for MAP1B and Tiam1, resembled Tau-1 distribution (Figure 1D). Magnification images showed in Figure 1C and 1E, support the codistribution of MAP1B and Tiam1 at the stage where axon is actively growing.

**Figure 2:**
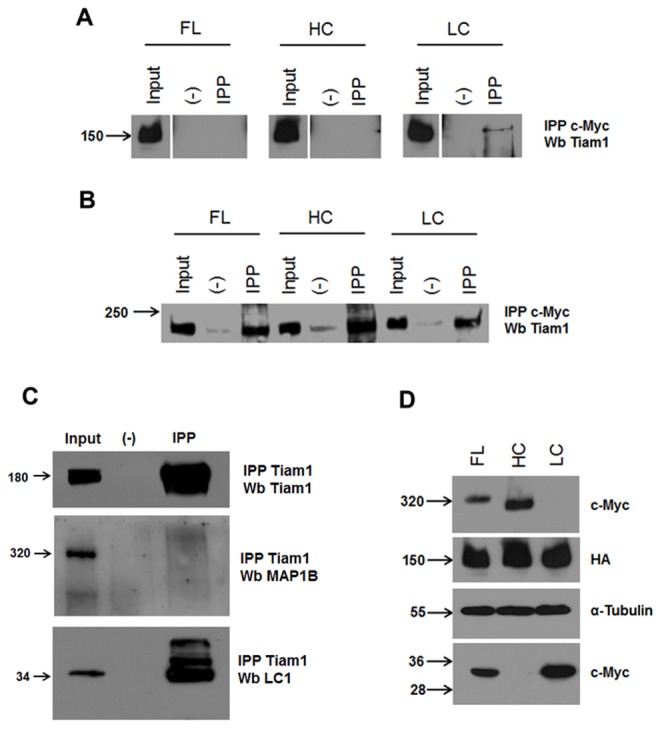
Tiam1 interacts with light chain 1 (LC1) of MAP1B. Overexpression of Myc-tagged MAP1B constructs in COS7 cells and N1E115 cells was analyzed by immunoprecipitation (IPP) with anti-myc antibodies followed by Western blotting (Wb) against endogenous Tiam-1 for N1E115 or Tiam-HA for COS7 cells. (A) myc-tagged MAP1B constructs expressed in COS7 cells are differentially recovered by HA-Tiam1. Note that only myc-LC1 subunit interact with Tiam1. (B) All myc-tagged MAP1B FL, HC and LC1 constructs expressed in N1E115 cells were able to recover Tiam1. (C) Only endogeneous N1E115 LC1 protein was recovered with Tiam1 under high stringent conditions (see materials and methods). (D) Myc-tagged MAP1B constructs expression in whole cell lysates. Full length (FL), Heavy chain (HC), and Light chain (LC) of MAP1B expressed in N1E115 (A) or COS7 (C) cells.

In order to characterize the molecular determinants involved in the MAP1B-Tiam1 interaction, we next expressed MAP1B using the tTA conditional expression system. Full-length (FL), heavy chain (HC) and light chain 1 (LC1) constructs tagged with a myc-epitope were transfected into either COS7 or. N1E115 neuroblastoma cells. Neuroblastoma cells contain endogeneous MAP1B expression, whilst COS7 cells do not. In order to elicit the expression of these constructs, a plasmid encoding the tetracycline trans-activator (tTA) was cotransfected with the MAP1B constructs. We then immunoprecipitated the MAP1B-myc tagged constructs and analyzed the presence of Tiam1, using an anti-Tiam1 antibody. Using this approach, we found that in a system devoided of endogenous MAP1B, such as COS7 cells, Tiam1 only interacted with the LC1 protein (Figure 2A). In contrast, Tiam1 was immunoprecipitated by all three MAP1B constructs (FL, HC and LC1, Figure 2B) in neuroblastoma cells. These results may reflect the formation of a complex composed by the endogenous LC1-Tiam1 dragging the heavy chain of MAP1B in neuroblastoma cells. In order to discriminate between direct or indirect binding of heavy chain to Tiam1, we performed another set of immunoprecipitation experiments where the washing steps were done in high stringency conditions to favor specific protein-protein interactions. Under these conditions, endogeneous Tiam1 immunoprecipitated LC1 from neuroblastoma cells, but failed to recover the heavy chain (Figure 2C). Figure 2D shows that a 320 kDa protein, which is recognized using an anti-c-myc antibody is expressed in the lane corresponding to the MAP1B-FL constructs. Analogously, the expression of both MAP1B-HC (∼280 kDa) and MAP1B-LC1 (∼34 kDa) constructs was detected using the same antibody, and as expected, MAP1B-HC migrates faster than MAP1B-FL (Figure 2D, lane HC). The expression of LC1 was also verified, since a 34 kDa protein was recognized by the anti-c-myc antibody (Figure 2D, lane LC). Interestingly, the MAP1B-FL construct gave rise to both a myc-tagged full-length and a myc-tagged-LC1 protein, suggesting that the MAP1B protein could be found in cells as the polyprotein precursor which could be proteolytically cleaved to release LC1 under basal experimental conditions. Altogether these results suggest that indeed the interaction between MAP1B and Tiam1 is established by the LC1 subunit of MAP1B.

### Tiam1 interacts with both the microtubule binding domain and actin binding domain of LC1

**Figure 3:**
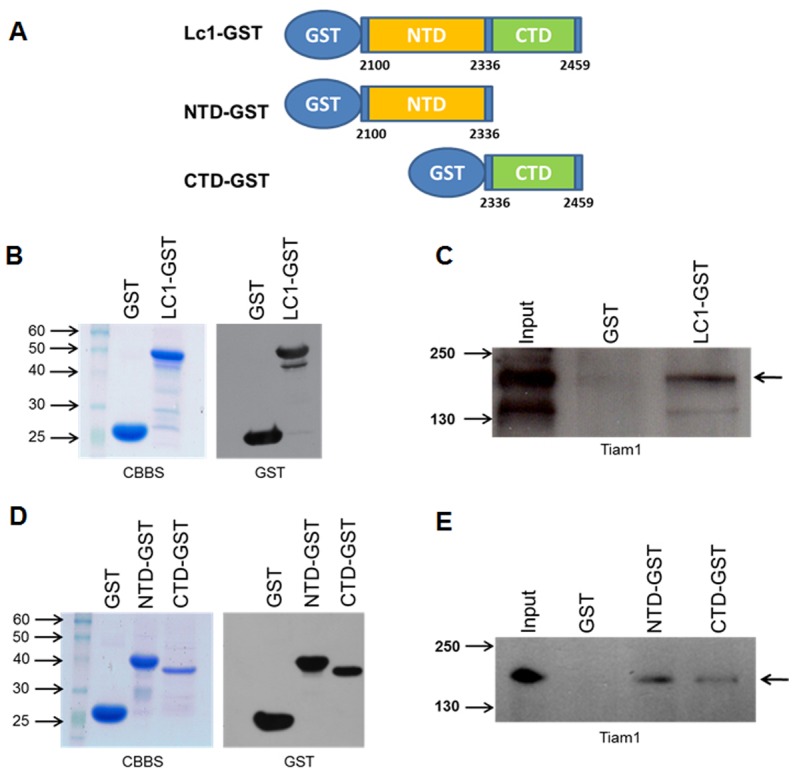
Tiam1 interacts with the N- and C-terminal domains of LC1. (**A**) Schematic representation of GST protein fused to LC1. Numbers refer to the amino acid positions in mouse MAP1B. **NTD**, N-terminal domain (microtubule binding domain); **CTD**, C-terminal domain (actin binding domain). (**B**) and (**D**) Coomassie brilliant blue stain (CBBS) of GST proteins used in pull down assays and immunoblot using anti-GST antibody. (**C**) and (**E**) Pull down assay using LC1-GST full length or isolated N- and C-terminal domains were incubated with fetal brain lysates. The bound protein was analyzed by immunoblotting with anti-Tiam 1 antibody. The arrows on the right-hand side of the images show the band corresponding to Tiam1.

In order to more-fully characterize the interaction between MAP1B and Tiam1, in the following experiments we specifically analyzed the interaction of Tiam1 with the LC1 subunit of MAP1B. We prepared GST-fusion proteins encoding the entire LC1 protein and constructs containing the N-terminal (microtubule binding domain) or C-terminal domains (actin binding domain) of the LC1 (Figure 3A). The recombinant, purified proteins were analyzed by Coomassie brilliant blue staining and with an anti-GST antibody in a western blot (Figure 3B and 3D). Both analyses demonstrate that recombinant proteins with the expected electrophoretic mobility were synthesized correctly. We used these constructs to pull down Tiam1 from fetal brain extracts. As indicated in Figure 3C, the GST-LC1 protein was able to specifically pull down Tiam1 from this extract. A control GST recombinant protein was used to discard unspecific binding of Tiam1 to the glutathione-sepharose GST protein (Figure 3C, GST). When we performed the pull down assay using the N-terminal (NTD-GST) and C-terminal (CTD-GST) domains of LC1, we found that both fragments were actually able to interact with Tiam1 (Figure 3E, NTD-GST and CTD-GST). Again the interaction did not exist when we incubated the protein extracts with a GST protein. Although the relative abundance of recovered Tiam1 seemed to be uneven when we used NTD-GST or CTD-GST (Figure 3E), this difference could be related with differences in the amount of recombinant protein produced (Figure 3D, compare NTD-GST with CTD-GST lanes). Indeed, in all the experiments producing LC1 fragments, we found that the CTD-GST construct was less efficiently expressed in bacteria.

### LC1 interacts with both N-terminal and C-terminal pleckstrin-homology domains (PH) of Tiam1

**Figure 4:**
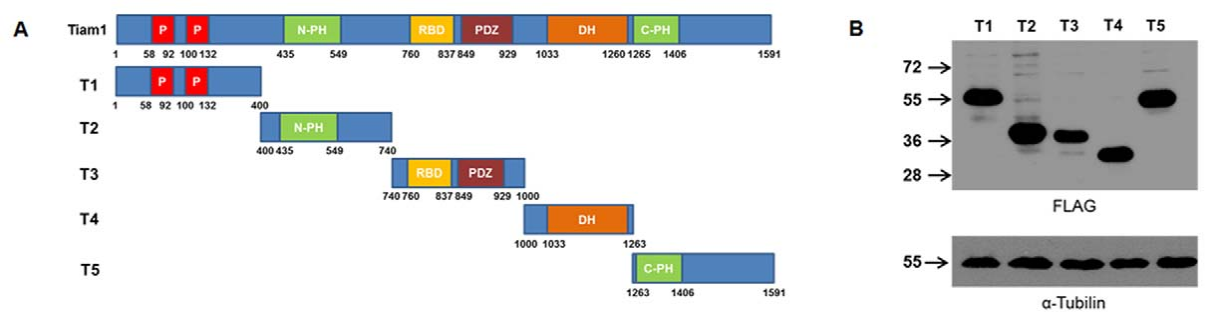
Deletion constructs of Tiam 1 containing a FLAG epitope at the N-terminal. (**A**) Schematic representation of Tiam1 and the deletion mutants. The numbers refer to the amino acid positions in mouse Tiam1. T1, N-terminal domain and PEST motif; T2, N-terminal pleckstrin domain (N-PH); T3, Ras binding domain (RBD) and PDZ domain; T4, Dbl-homology domain (DH) and T5 C-terminal pleckstrin domain (C-PH). (**B**) immunoblotting showing the expression of the FLAG-Tiam1 constructs in COS-7 cells.

**Figure 5:**
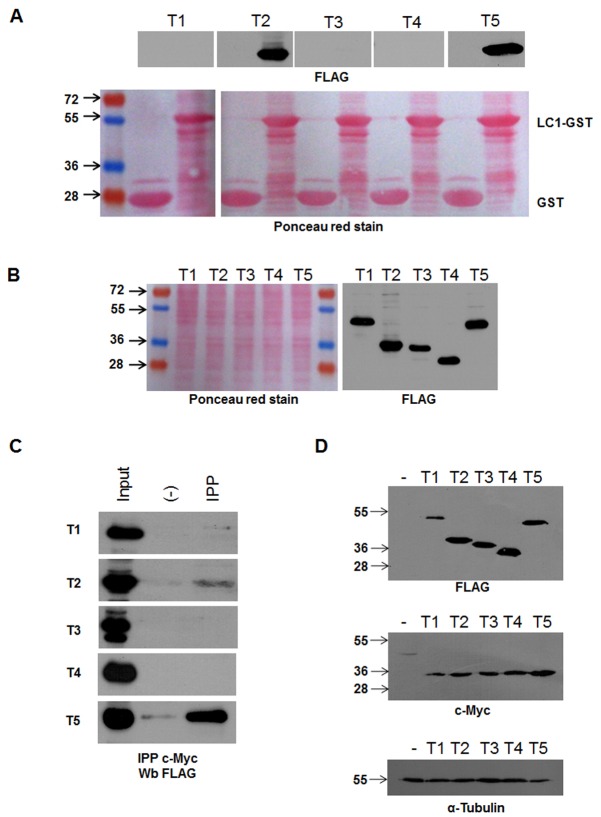
Mapping of the region in Tiam 1 that is required for binding to LC1. LC1-GST immobilized beads were incubated with cell lysates of COS7 cells expressing the FLAG-Tiam1 constructs. As indicated in (**A**), only domains containing the pleckstrin domain of Tiam 1 (T2 and T5) interact with LC1-GST, as visualized by immunoblotting using an anti-FLAG antibody. (**B**) Panel shows the total lysate of COS7 cells expressing each of the Tiam1 fragment proteins used for the pull down assays. (**C**) N-PH (T2) and C-PH (T5) interacted with LC1 in immunoprecipitation assays of Tiam1 fragments with LC1-Myc. Proteins were expressed in COS7 cells (**D**) (input of immunoprecipitation reaction).

In order to determine which region of Tiam1 is involved in the interaction with MAP1B-LC1, we then prepared different constructs of the Tiam1 protein for expression in cellular systems. For such a purpose, we divided the cDNA encoding full-length Tiam1 of mouse embryonic brain mRNA into five discrete regions (T1–T5), as indicated in Figure 4A. All the constructs contained an N-terminal FLAG-epitope. The expression of these constructs was verified in transfected COS7 cells (Figure 4B), and peptides of the expected size were detected with an anti-FLAG antibody. The protein extracts derived from COS7 cells expressing Tiam1 constructs T1 to T5 were pulled down using the GST-LC1 recombinant protein. Using this approach, we found that LC1 only interacted with the constructs T2 and T5, which correspond to the N-terminal and C-terminal PH domains of Tiam1, respectively (Figure 5A). The differential interaction was not due to problems in the expression of constructs (Figure 5B) or the amount of LC1-GST protein used in the pull down assays (Figure 5A, see Ponceau red staining in the lower panel). These results suggest that PH domains of Tiam1 were responsible for the interaction with LC1. In order to confirm these findings, we coexpressed each Tiam1 construct (T1 to T5) with LC1-myc in COS7 cells. Using an anti-c-myc antibody, we immunoprecipitated LC1 and analyzed the co-immunoprecipitation of each Tiam1 fragment using an anti-FLAG antibody. Figure 5C shows that similarly to pull down assay, myc-LC1 was able to interact with T2 and T5 Tiam1 constructs. Figure 5D shows that the interactions detected in the study were not due to the differential expression of the Tiam1 (T2 to T5) or LC1 constructs in COS7 cells.

**Figure 6:**
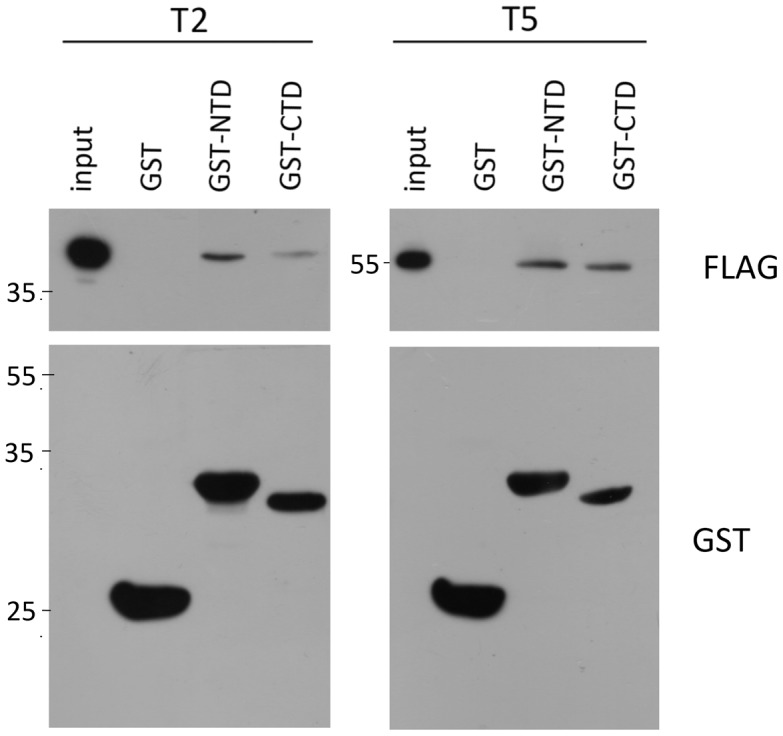
N-PH and C-PH domains of Tiam 1 interact with both domains of the light chain 1 (LC1) of MAP1B. Pull down assays using GST-fusion proteins of LC1, indicated by western blots using anti-GST antibody (bottom of each panel), were incubated with lysates of COS7 cells expressing T2 (N-PH) or T5 (C-PH). Both fragments of Tiam1 (T2 and T5) interacted with NTD and CTD of LC1.

The interaction of these Tiam1 T2 and T5 fragments with the NTD and CTD of LC1 was then analyzed. The protein extracts derived from COS7 cells expressing Tiam1 constructs T2 and T5 were pulled down using NTD-GST and CTD-GST fusion proteins of LC1. After the pull down assay, we found that both T2 and T5 fragments interacted with the N-terminal and C-terminal domain of LC1, suggesting that full length LC1 is required to establish the interaction between Tiam1 and MAP1B (Figure 6).

### LC1 increase the Rac1 activity

**Figure 7:**
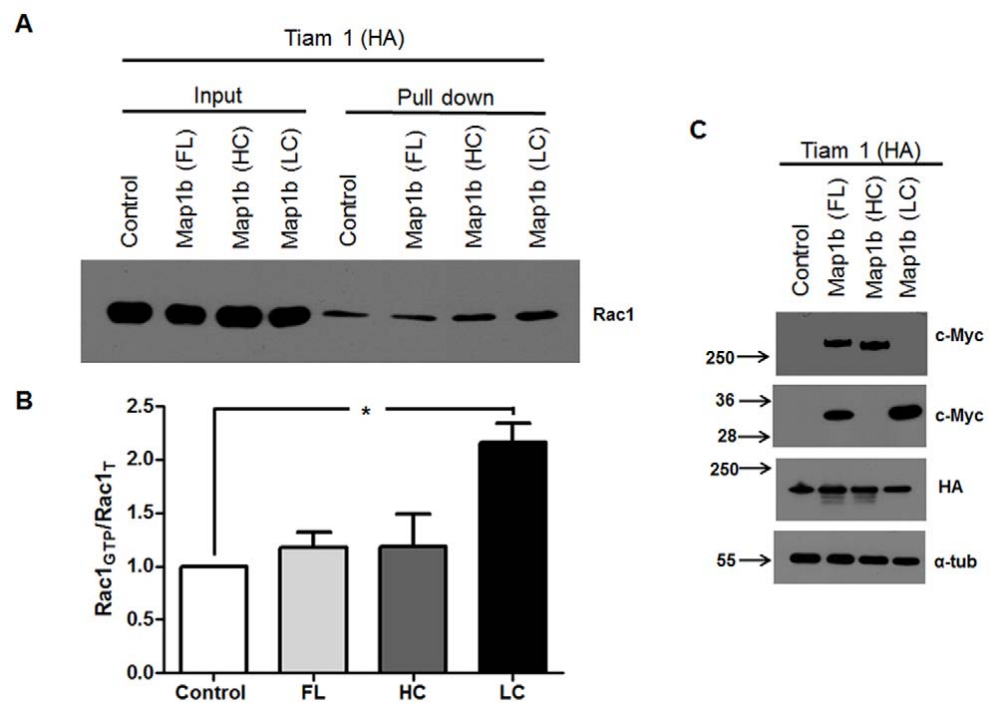
LC1 of MAP1B but not HC or FL increases Rac1 activity. (**A**) Western blot against Rac1 showing that total lysate protein obtained from the COS7 cells expressing Tiam1 (C1199 HA) and myc-tagged LC1 contain around two-fold more Rac1-GTP than equivalent samples obtained from COS7 cells expressing Tiam1 (C1199 HA) and the HC or FL of MAP1b. Total Rac1 and α-tubulin were used as loading controls. (**B**) Quantitative analysis confirmed a significant increase (*p<0.05) in Rac1 activity in the presence of the LC1 domain of MAP1B. (**C**) Panel shows the total lysate of COS7 cells expressing each of the proteins used for pull down assay activity of Rac1.

Having shown that Tiam1, a GEF of Rac1, and LC1 interact *in vitro* (N1E115 and COS7 cells) and the brain (pull down), we were interested in determining the effect of each MAP1B construct upon Rac1 activity. We expressed each MAP1B construct (FL, HC, and LC1) in neuroblastoma N1E115 cells, and measured the activity of Rac1. For such a purpose, we used an immobilized GST-fusion protein corresponding to the effector of Rac1, the p21-binding domain (residues 67–150) of murine PAK1, which binds Rac1-GTP (active form) with high affinity but not Rac1-GDP (inactive form) in a pull down assay. Bound proteins were separated by SDS-PAGE and immunoblotting was performed with anti-Rac1. The presence of endogenous MAP1B masks the effect of the isolated fragments (HC and LC1) upon Rac1 activity (Figure S1). Therefore, we performed the same experiment in cells lacking endogeneous MAP1B (COS7) and assayed for Rac1 activity. The highest Rac1 activity was found when Tiam1 was coexpressed with LC1, reinforcing the notion that the LC1-Tiam1 interaction elicits Rac1 activity (Figure 7A). Quantitative analyses from three independent experiments showed that LC1 expression doubled the activity of Rac1 (p value<0,05, ANOVA one-way), while Rac1 activity after expression of FL and HC did not differ from untransfected controls (Figure 7B). This difference in the activity of the Rac1 was not due to problems in the expression of constructs (Figure 7C). The results obtained in neuroblastoma cells support that endogenous MAP1B heavy and light chain 1, can assemble a protein complex with expressed myc-HC and myc-LC1 construct, making not possible to distinguish the real contribution for each fragment toward Rac1 activity. Nonetheless, we previously showed that neurons lacking MAP1B, show decreased Rac1 activity [20]. Altogether, these results indicate that LC1 of MAP1B interacts with both PH domains of Tiam1, increasing Rac1 activity. Additionally, it implies that the HC might serve as a regulatory subunit in the macromolecular complex.

## Discussion

The establishment and maintenance of neuronal polarity depends on a tight coordination of cytoskeletal rearrangements and directed membrane trafficking [5], [39]. It has been shown that Tiam1-dependent localized activation of Rac1 contributes to the rapid elongation of the nascent axon [18]. Recently, we showed that neurons lacking MAP1B (an axonal microtubule-associated protein) displayed decreased Rac1 and Cdc42 activities and concomitantly increased RhoA activity [20]. The abnormal activities of these small Rho GTPases found in neurons lacking MAP1B contribute to a phenotype characterized by inhibited axonal elongation [28]. Interestingly, a similar mechanism seems to be involved in the generation and maintenance of dendritic spines in adult neurons [21]. A working model, therefore, establishes that the interaction of MAP1B with Tiam1 promotes the spatiotemporal activation of Rac1, supporting a role for MAP1B in the cross-talk mechanisms of actin and microtubules during axonal elongation [20]. The MAP1B-Tiam1 interaction is specific, since tau (another axonal MAP) does not interact with Tiam1 [21].

MAP1B is encoded as a single polyprotein (full length MAP1B) with one cleavage site located toward the C-terminal of the molecule, leading to the production of heavy chain (HC) and light chain 1 (LC1) fragments [40]. Both fragments (HC and LC1) possess microtubule and actin binding domains [29], [30], [36], [37]. In the present work, we characterized the domains involved in the MAP1B-Tiam1 interaction, and determined the fragment of MAP1B responsible for Tiam1-dependent Rac1 activation. We showed that LC1 is sufficient to promote Tiam1-dependent Rac1 activation. Moreover, we demonstrated that full length MAP1B seemed to have a regulatory role, since it was not able to activate Rac1. Using a non-neuronal heterologous system, we showed that LC1 specifically interacted with Tiam1. Interestingly, an analogous experiment in neuronal cells showed that both HC and LC1 could interact with Tiam1. However, the interaction between heavy chain and Tiam1 was prevented by more stringent immunoprecipitation conditions, suggesting that heavy chain was indeed recruited bound to the LC1-Tiam1 complex. Previous studies indicate that brain extracts have considerably more LC1 than HC (8∶1) and that this ratio decreases to 2∶1 in purified microtubule samples [40]. Accordingly, recombinant HC and LC1 proteins may assemble with endogenous fragments present in neuronal cells, leading to misleading interpretations. We identified the pleckstrin homology domain (N-PH and C-PH) of Tiam1 as involved in the interaction with LC1. Mouse Tiam1 is a 1591 amino acid protein with discrete and well-known domains. The N-terminal domain of the protein has a myristoylation site responsible for plasma membrane binding [41]. It also contains two PEST domains which are believed to be responsible for targetting the protein for rapid degradation [42]. The N-terminal PH domain (N-PH) present in Tiam1 (and Tiam2) is critical for Tiam1 membrane localization [41], [43]. Recently, the N-PHCCEx domain has been crystalized, defining two basic contact zones at the CC and Ex-subdomains that bind to specific acidic motifs present in membrane proteins such as CD44 and ephrin B [44]. In contrast, the N-PH subdomain is negatively charged, with a groove between β3–β4 and β5–β6 loops that may explain the interaction with LC1 which possesses a well-defined positively-charged motif in the microtubule binding domain [45]. It is worth noting that the isoelectric points (IP) of the MAP1B subunits are different. While the LC1 IP is 9.23, full length MAP1B displays IP of 4.76; the latter is therefore, less likely to interact with Tiam1. The central domain of Tiam1 is characterized by the presence of a Ras-binding domain (RBD) [46] and a PDZ domain [47]. The C-terminal domain of Tiam1 contains the characteristic Dbl homology (DH) and pleckstrin homology (C-PH) tandem domain, present in all members of the Dbl family of GEFs (reviewed by [48], [49]). *In vitro*, the isolated DH domain elicit nucleotide exchange in Rac1, but the C-PH domain is necessary to promote Rac1 activity *in vivo*. Truncations of the C-PH domain eliminate the ability of Tiam1 to induce membrane ruffles in cells [50]. The presence of two different PH domains in Tiam1 is related with the fact that C-PH differs from the canonical DH-PH domain. Specifically, the C-PH domain has a lower affinity for phosphoinositides and is not involved in membrane localization of the protein [41], [43], [51]. However, a possible role for this C-PH domain is to promote protein-protein contacts with Tiam1 ligands that stimulate Rac1 activity. Structural studies show that the interaction between Rac1 and Tiam1 occurs primarily through switch 1 (residues 25–39) and switch 2 (residues 57–75) of Rac1 and the CR-1 and CR-3 regions of the Tiam1 DH domain [52]. In this complex, the C-terminal of the DH domain interacts with the Rac1 and C-PH domain, suggesting that structural stabilization of Tiam1 may be dependent of the DH-CPH tandem domain. In this context, the increase of Rac1 activity observed in cells expressing Tiam1 and LC1 suggests that the interaction of both PH domains of Tiam1 with LC1 is involved in this activation. Two possible molecular mechanisms can be envisioned. First, the interaction of LC1 and cytoskeleton elements at subcortical domains may recruit Tiam1 promoting Rac1 activation. A second scenario could be related with a direct modulation of MAP1B upon Rac1-Tiam1 interaction, owing to the binding to PH domains in Tiam1. These possibilities are not mutually exclusive, since we were not able to detect significant differences in the affinity of either LC1 or NTD/CTD domains for Tiam1 PH domains. Other cellular mechanisms involving post-translational modifications of Tiam1 can not be ruled out. Tiam1 proteins can be phosphorylated by Rho kinases, in a Rho A-dependent manner, leading to inactivation of Rac1 signaling [53]. In spite of the molecular mechanism regulating the interaction of MAP1B and Tiam1, these results support a novel role for MAP1B, linking the dynamic changes occurring in both microtubules and actin microfilaments.

## Supporting Information

Figure S1 **The presence of endogenous MAP1B in N1E115 cells masks the effects of the myc-tagged LC1 over the Rac1 activity.** Pull down assay to measure Rac1 activity in N1E115 cells expressing Tiam1 (C1199 HA) and myc-tagged MAP1B fragments (FL, HC and LC1), display similar levels of Rac1-GTP.
(TIF)
