# Supplementary figures and images for: The Light Chain 1 Subunit of the Microtubule-Associated Protein 1B (MAP1B) Is Responsible for Tiam1 Binding and Rac1 Activation in Neuronal Cells
Source: PLoS One. 2012 Dec 27;7(12):e53123. doi: 10.1371/journal.pone.0053123 (PMC3531375; doi:10.1371/journal.pone.0053123)

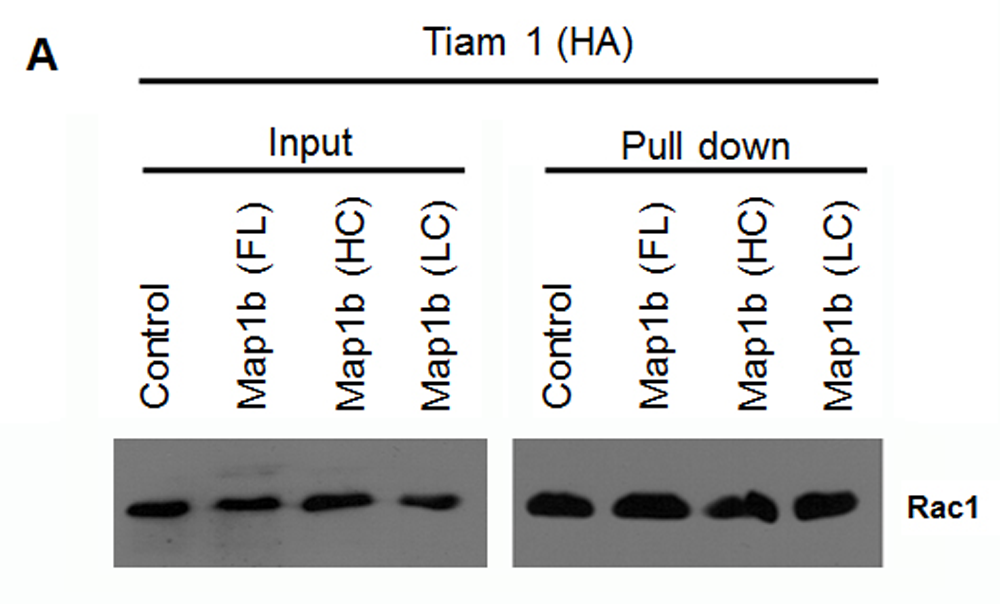

Supplement: Figure S1 — The presence of endogenous MAP1B in N1E115 cells masks the effects of the myc-tagged LC1 over the Rac1 activity. Pull down assay to measure Rac1 activity in N1E115 cells expressing Tiam1 (C1199 HA) and myc-tagged MAP1B fragments (FL, HC and LC1), display similar levels of Rac1-GTP. (TIF) [file pone.0053123.s001.tif]
